# Contribution of New Adenomatous Polyposis Predisposition Genes in an Unexplained Attenuated Spanish Cohort by Multigene Panel Testing

**DOI:** 10.1038/s41598-019-46403-5

**Published:** 2019-07-08

**Authors:** Víctor Lorca, Daniel Rueda, Lorena Martín-Morales, María Jesús Fernández-Aceñero, Judith Grolleman, Carmen Poves, Patricia Llovet, Sandra Tapial, Vanesa García-Barberán, Julián Sanz, Pedro Pérez-Segura, Richarda M. de Voer, Eduardo Díaz-Rubio, Miguel de la Hoya, Trinidad Caldés, Pilar Garre

**Affiliations:** 1 Laboratorio de Oncología Molecular, Hospital Clínico San Carlos, IdISSC, CIBERONC, Madrid, Spain; 2 Laboratorio de Cáncer Hereditario, Servicio de Bioquímica, i + 12, Hospital 12 de Octubre, Madrid, Spain; 3 Servicio de Anatomía Patológica, Hospital Clínico San Carlos, Madrid, Spain; 4 Department of Human Genetics, Radboud University Medical Center, Nijmegen, The Netherlands; 5 Servicio de Aparato Digestivo, Hospital Clínico San Carlos, Madrid, Spain; 6 Servicio de Oncología Médica, Hospital Clínico San Carlos, CIBERONC, Madrid, Spain

**Keywords:** Cancer genetics, Genetic testing, Colorectal cancer

## Abstract

Attenuated adenomatous polyposis (AAP) is a heterogeneous syndrome in terms of clinical manifestations, heritability and etiology of the disease. Genetic heterogeneity and low penetrance alleles are probably the best explanation for this variability. Certainly, it is known that *APC* and *MUTYH* are high penetrance predisposition genes for adenomatous polyposis, but they only account for 5–10% of AAP. Other new predisposition genes, such as *POLE*, *POLD1*, *NTHL1*, *AXIN2* or *MSH*3, have been recently described and have been associated with AAP, but their relative contribution is still not well defined. In order to evaluate the genetic predisposition to AAP in a hospital based population, germline DNAs from 158 AAP subjects were screened for genetic variants in the coding regions and intron-exon boundaries of seven associated genes through a next-generation sequencing (NGS) custom gene panel. Splicing, segregation studies, somatic mutational screening and RNA quantitative expression assays were conducted for selected variants. In four of the probands the adenoma susceptibility could be explained by actionable mutations in *APC* or *MUTYH*, and one other patient was a double carrier of two truncating variants in both *POLE* and *NTHL1*. Furthermore, 16 additional patients harbored uncertain significance variants in the remaining tested genes. This report gives information about the contribution of the newly described adenomatous polyposis predisposition genes in a Spanish attenuated polyposis cohort. Our results highly support the convenience of NGS multigene panels for attenuated polyposis genetic screening and reveals *POLE* frameshift variants as a plausible susceptibility mechanism for AAP.

## Introduction

Attenuated adenomatous polyposis (AAP) is usually defined by the presence of more than 10 and less than 100 adenomas along the large intestine and/or rectum. It is a highly heterogeneous syndrome in terms of polyposis severity, family history, and lifetime risk of colorectal cancer (CRC)^1–3^. Adenoma burden can be low or mild, it may be accompanied by hyperplastic or serrated polyps, the polyposis diagnosis age is variable and later than classical forms, and CRC risk can range from 40% to 80% depending on the adenoma burden. A positive family history is frequently absent, but horizontal or vertical inheritance patterns are also observed. The diffusion of early detection CRC screening programs, together with the improvement of colonoscopy techniques, has given rise to an increase in the detection of patients with multiple colorectal adenomas. These patients are referred to the oncogenetic counseling units for genetic testing in order to find an explanation to their adenoma susceptibility that helps in the understanding of the disease and makes possible a satisfactory genetic counseling. Nowadays*, APC* (MIM *611731) and *MUTYH* (MIM *604933) are the two main clinical actionable predisposition genes for adenomatous polyposis^3^. That means prevalence and cancer risk estimations are well-defined, allowing an accurate genetic counseling and effective high risk monitoring programs for carriers. They both together explain the vast majority of classical adenomatous polyposis (>100 adenomas), but they are only able to explain a small fraction of AAP^2^. In fact, in our hospital settings, we are not able to explain more than 5–10% of the patients referred to our laboratory after the routine genetic tests. Therefore, the identification of the underlying susceptibility causes of those unexplained AAP cases is a priority in our laboratory.

Recently, other adenomatous polyposis predisposition genes, such as *POLE*^4^, *POLD1*^4^, *NTHL1*^5^, *MSH3*^6^ *and AXIN2*^7^ (MIM *174762, *174761, *602656, *600887 and *604025, respectively), have been described. However, they are still not well characterized and only a few reports have attempted to estimate the extent to which some of these genes, individually, are involved in different CRC populations^8–13^. Therefore, they are still poorly implemented in the daily clinical practice.

Under this scenario of high genetic heterogeneity, the use of next-generation sequencing (NGS) gene panels for the diagnosis of hereditary AAP seems to be the best choice. Aiming to delve into the genetic study of unexplained AAP, we have screened the whole coding sequences and intron-exon boundaries of *APC*, *MUTYH*, *POLE*, *POLD1*, *NTHL1, MSH3* and *AXIN2* in a cohort of AAP collected at our oncogenetic counseling unit. The main objective of this work is to determine the contribution of the AAP associated genes in an unexplained AAP cohort.

## Results

### Clinical features of the study cohort

A clinical description of the study cohort is summarized in Table 1. The study cohort consisted of 158 AAP patients, coming from the Oncogenetic Counseling Units of Hospital Clínico San Carlos and Hospital 12 de Octubre, in Madrid. The average polyposis diagnosis age was 62.9 (ranged from 33 to 80) and the average polyp burden 31.2 (from 10 to 100). Detailed clinical description of participants is given in Suppl. Table 1.

**Table 1:** Clinicopathological characteristics of the study cohort.

	AAP COHORT
N	158
% males (n)	73.4 (116)
Dx Age; average (range)	62.9 (33–80)
N adenoma; average (range)	31.2 (10–100)
% hyperplastic polyp detection (n)	44.3 (70)
% dominant inheritance pattern (n)	22.2 (35)
% recessive inheritance pattern (n)	30.4 (48)
% isolated case (n)	47.5 (75)
% full-blown AAP^†^ (n)	83.5 (132)

^†^Full-blown AAP = subjects with more than 20 adenomas or 10 synchronous adenomas.

### Germline DNA screening

After genetic panel screening, the average read depth per sample was 895 reads, with a minimum count of 326 and a maximum of 4616. However, two regions showed reiterative low coverage, so they were reanalyzed by high resolution melt (HRM) analysis, NM_000038.4: exon 13 (*APC*) and NM_00128425.1: exon 15 (*MUTYH*). Once the screening was completed, 28 variants located in six genes were validated in 24 patients (Table 2).

**Table 2:** Validated variants and classification according to ACMG-SHERLOC criteria^42^.

GENE	ID	CHANGE (HGVS)	rs ID	gnomAD^†^	PD^‡^	CL^§^
*APC*	*3*	c.266C > G p.(Ser89*)		nd	NA	5
	9	c.147_150del p.(Lys49Asnfs*20)		nd	NA	5
	34	c.1966C > G p.(Leu656Val)	rs577466163	0	ARM	3
	133	c.7399C > A p.(Pro2467Thr)	rs372305287	1e-04	APC basic	3
	136	c.8501A >C p.(His2834Pro)		nd	EB1_hDLG	3
	139	c.1240C > G p.(Arg414Cys)	rs137854567	nd	—	3
*AXIN2*	55	c.2141G > A p.(Arg714Gln)	rs762872515	0	—	3
	79	c.203G > A p.(Arg68Gln)	rs138056036	2e-04	AXIN1_TNKS	3
*MUTYH*	35	c.1187G > A p.(Gly396Asp)	rs36053993	4.8e-03	NUDIX 4	5
	35	c.739C > G p.(Arg247Gly)		nd	ENDO3c	4
	37	c.1187G > A p.(Gly396Asp)	rs36053993	4.8e-03	NUDIX 4	5
	38	c.1187G > A p.(Gly396Asp)	rs36053993	4.8e-03	NUDIX 4	5
	61	c.667A > G p.(Ile223Val)	rs200872702	3.4e-04	ENDO3c	3
	89	c.1187G > A p.(Gly396Asp)	rs36053993	4.8e-03	NUDIX 4	5
	89	c.1510_1517delinsCCAACAGCCA p.Thr504Profs*68		nd	NA	5
	99	c.1187G > A p.(Gly396Asp)	rs36053993	4.8e-03	NUDIX 4	5
*NTHL1*	16	c.527T > C p.(Ile176Thr)	rs1805378	2.2e-03	ENDO3c	3
	75	c.856G > A p.(Gly286Ser)	rs139309757	3.2e-05	ENDO3c	3
	82	c.527T > C p.(Ile176Thr)	rs1805378	2.2e-03	ENDO3c	3
	83	c.268C > T p.(Gln90*)	rs150766139	2.0e-03	NA	5
*POLD1*	116	c.2007-5C > T	rs199506387	2.5e-04	NA	1
	118	c.520C > T p.(Arg174Trp)	rs749334182	2.7e-05	POLBc-exo	3
	152	c.2052G > C p.(Gln684His)	rs144143245	6.3e-04	POLBc-pol	3
	157	c.1465G > A p.(Val489Met)	rs753244422	3.3e-05	POLBc-exo	3
*POLE*	16	c.3857G > A p.(Arg1286His)	rs771823596	5e-05	low complex	3
	21	c.6716C > T p.(Ala2239Val)	rs190813054	8e-06	—	2
	83	c.141delG p.(Phe48Leufs*6)	rs761329565	0	NA	4
	147	c.198G > A p.(Met66Ile)	rs764962999	0	—	3

All variants were heterozygous. ^†^GnomAD (08/06/2018): European (Non-Finish) population frequencies (Exome + Genome). ^‡^PD = Protein domain from SMART protein database: NA = not applicable. ^§^CL: variant classification: 1 = benign; 2 = likely benign; 3 = uncertain significance; 4 = likely pathogenic; 5 = pathogenic. Reference sequences: *APC:* NM_000038, NP_000029; *AXIN2*: NM_004655, NP_004646; *MUTYH*: NM_001128425, NP_036354; *NTHL1*: NM_002528, NP_002519; *POLD1*: NM_001256849, NP_001121897; *POLE*: NM_006231, NP_006222.

Eight patients harbored pathogenic variants (class-5): Two patients harbored class-5 variants in *APC*, one patient was a biallelic carrier of two class-5 variants, another was *MUTYH* biallelic carrier of a class-5 and a class-3 variants, and three patients were monoallelic *MUTYH* class-5 carriers. Finally, one patient was a double carrier of two deleterious variants, in *POLE* and *NTHL1*. The remaining sixteen patients harbored class-3 (Table 2).

### Reclassification of variants

After the splicing, segregation and somatic analyses, it was possible to confirm two *MUTYH* variants (c.739C > G; p.(Arg247Gly) and c.1510_1517delinsCCAACAGCCA; p.Thr504Profs*68) as likely pathogenic (class-4) and pathogenic (class-5), respectively, and to reclassify the variant *POLD1:* c.2007-5C > T as benign (class-1) and *POLE*: c.6716C > T p.(Ala2239Val) as likely benign (class-2) (Table 2, Suppl. Table 3A,B). In the end, 11 out of 28 variants were classified as class-5 or -4, two variants were classified as class-1 or -2, discarding their involvement in the AAP predisposition, and 15 variants remained as uncertain significance (class-3) variants.

#### *MUTYH*:c.739C > G p.(Arg247Gly)

**Figure 1:**
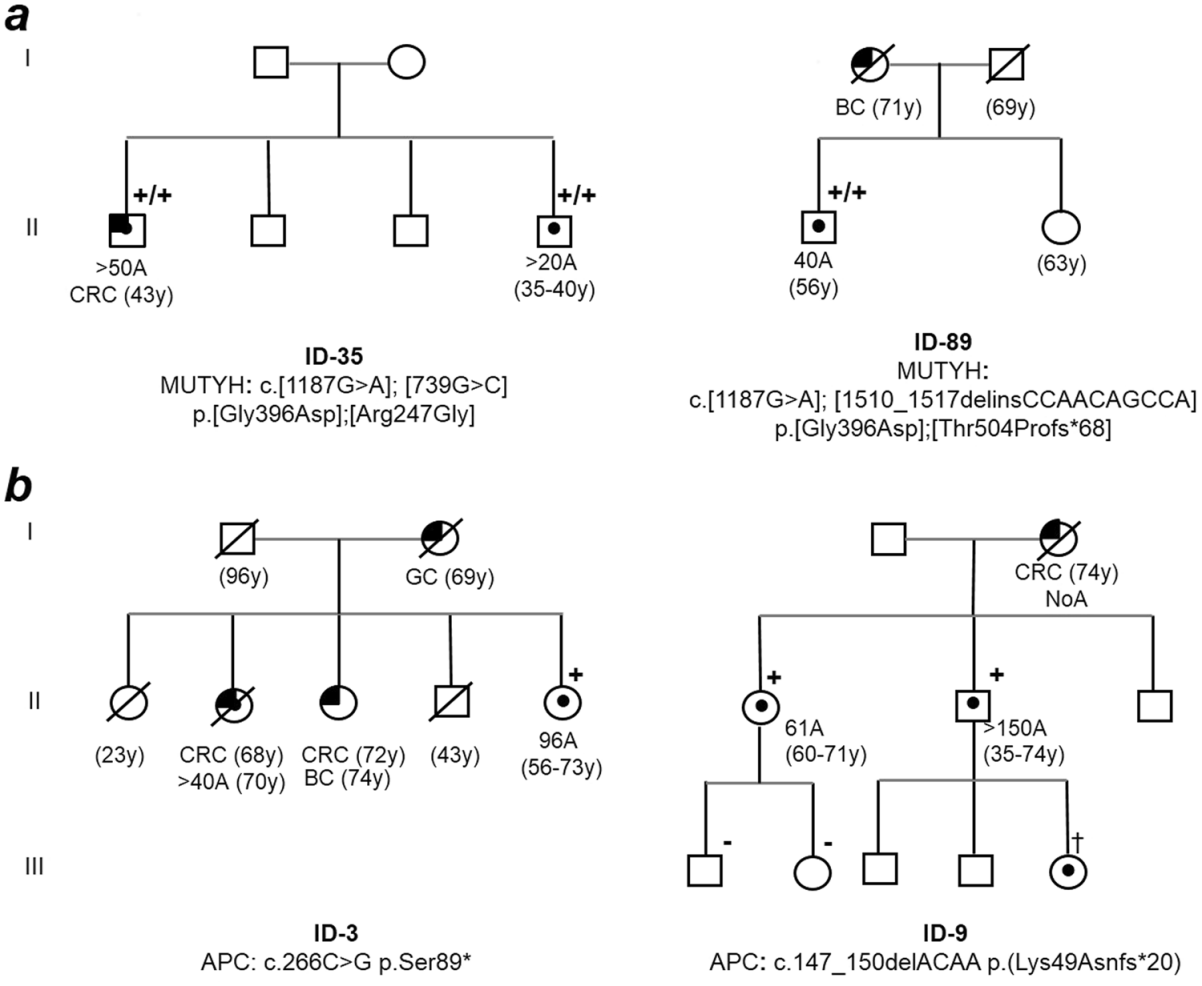
Pedigrees of the explained polyposis cases. (**a**) *MUTYH* biallelic mutations carrier families. (**b**) *APC* mutations carrier families. ^†^polyposis diagnosis after family mutation identification. Black square = cancer; black circle = >10 adenomas; ^+=^variant carrier; ^−^non carrier; ^+/+^biallelic carrier; GC = gastric cancer; CRC = colorectal cancer; BC = breast cancer; y = years; A = adenomas.

*MUTYH* variant c.739C > G; p.(Arg247Gly) was detected in *trans* (Suppl. Fig. 1a) with c.1187G > A; p.(Gly396Asp) in a male with more than 20 adenomas at the age of 37. One of his brothers was also a carrier of both variants and presented more than 50 adenomas and CRC at the age of 41(Fig. 1a). The variant is located in the hMSH6 binding domain, and missense mutations located in this domain have been shown to affect the A/8-oxoG binding and glycosylase activities^14^. Adenomatous tissue from the proband was screened for somatic mutations and G > T changes were found in *APC*, *KRAS, TP53 and MAP2K* (Table 3, ID 35). As it is well known, adenomas and tumors coming from *MUTYH* biallelic carriers show a deficiency in the 8-oxo-hidroxyguanine repair system, leading to an increase in the G > T mutation rate, frequently in *APC* and *KRAS*^15^. Thus, c.739C > G; p.(Arg247Gly) was reclassified as class-4 (Suppl. Table 3).

**Table 3:** Somatic mutations.

IDP^†^	IDS^‡^	HIST^§^	GENE	CHANGE	TRIPLET	CHANGE (HGVS)	% ALT^¶^	MET^††^
83	**AD5**	LDTA	*APC*	C > C/T	G**C**G > G**T**G	c.4348C > T p.(Arg1450*)	5.7	TST
			*APC*	C > C/A	T**C**A > T**A**A	c.4381G > T p.(Glu1461*)	6.6	TST
			*CTNNB1*	C > C/T	T**C**T > T**T**T	c.134C > T p.(Ser45Phe)	12.9	TST
	**AD4**	LDTA	*ARID1A*	C > C/T	A**C**A > A**T**A	c.514C > T p.(Gln172*)	26	MIP
			*APC*	C > C/T	G**C**G > G**T**G	c.2626C > T p.(Arg876*)	8	MIP/TST
			*APC*	AAG > AAG/A		c.3924_3925delAG p.(Glu1309Lysfs*5)	8.8	MIP/TST
			*APC*	GA > GA/G		c.3926delA p.(Lys1310Argfs*11)	21	MIP/TST
	**AC1**	ACH0	*APC*	C > C/A	T**C**T > T**A**T	c.3916G > T p.(Glu1306*)	32	MIP/TST
			*RNF43*	C > C/T	C**C**A > C**T**A	c.745C > T p.(Gln249*)	12	MIP
			*NRAS*	C > C/T	A**C**C > A**T**C	c.38G > A p.(Gly13Asp)	4.5	TST
	**AD1**	LDTA	*APC*	C > C/T	G**C**G > G**T**G	c.1660C > T p.(Arg554*)	15	MIP
			*APC*	C > C/A	T**C**A > T**A**A	c.4189G > T p.(Glu1397*)	13	MIP
	**AD3**	LDTA	*APC*	C > C/A	T**C**T > T**A**T	c.3916G > T p.(Glu1306*)	34.8	TST
35	**AD1**	LDTA	*APC*	CC > CC/TA		c.4479_4480delGGinsAT p.(Glu1494*)	18.3	TST
		LDTA	*MAP2K1*	C > C/A	G**C**T > G**A**T	c.171G > T p.(Lys57Asn)	16.8	TST
		LDTA	*TP53*	C > C/A	T**C**T > T**A**T	c.859G > T p.(Glu287*)	19.1	TST
	**AD2**	LDTA	*APC*	C > C/A	G**C**A > G**A**A	c.4230C > A p.(Cys1410*)	7.6	TST
		LDTA	*APC*	C > C/T	G**C**G > G**T**G	c.2626C > T p.(Arg876*)	6.8	TST
		LDTA	*KRAS*	C > C/A	C**C**A > C**A**A	c.37G > T p.(Gly13Cys)	6.7	TST
37	**AD1**	LDTA	*APC*	C > C/−		c.3955delC p.(Pro1319Leufs*2)	15.7	TST
		LDTA	*TP53*	C > C/T	G**C**G > G**T**G	c.524G > A p.(Arg175His)	11.5	TST
38	**AD1**	TA LDTA LD	*MAP2K1*	C > C/A	G**C**T > G**A**T	c.171G > T p.(Lys57Asn)	4.6	TST
	**AD2**	HDTVA	*APC*	T > T/−		c.4233delT p.(Ser1411Argfs*4)	37.1	TST
61	**AD1**	LDTA	*CDH1*	−>−/A		c.1885_1886insA p.(Leu630Thrfs*33)	7.2	TST
		LDTA	*CTNNB1*	T > T/C	G**T**A > G**C**A	c.121A > G p.(Thr41Ala)	12.1	TST
		LDTA	*NRAS*	C > C/T	G**C**A > G**T**A	c.360G > A p.(Leu120 = )	50.6	TST
99	**AD1**	LDTA	*APC*	C > C/T	G**C**G > G**G**G	c.4348C > T p.(Arg1450*)	35.7	TST
		LDTA	*FBXW7*	C > C/T	T**C**G > T**T**G	c.1436G > A p.(Arg479Gln)	35.5	TST
		LDTA	*KRAS*	C > C/T	C**C**A > C**T**A	c.34G > A p.(Gly12Ser)	45.6	TST
89	**AD1**	LDTA	*KRAS*	C > C/A	C**C**A > A**C**A	c.34C > A p.(Gly12Cis)	>1.5	TH-K
	**AD2**	LDTA	*KRAS*	C > C/A	C**C**A > A**C**A	c.34C > A p.(Gly12Cis)	>1.5	TH-K

Somatic mutations detected in adenomas analyzed. ^†^IDP = Pacient ID; ^‡^IDS = Sample ID: AD = adenoma AC = adenocarcinoma; ^§^HIST = Histology: LDTA = Low grade dysplastic tubular adenoma, ACH0 = Adenocarcinoma arising from adenoma Haggitt 0, HDTVA = High grade dysplastic tubulovillous adenoma; ^¶^% ALT = percentage of altered allele; ^††^MET = Method: TST = TruSight Tumor 26 panel (Illumina); MIP = single molecule Molecular Inversion Probe; TH-K = Therascreen®KRAS. Reference sequences: *APC*: NM_000038, NP_000029; *CTNNB1*: NM_001098210, NP_001091680; *ARID1A*: NM_006015, NP_006006; *RNF43*: NM_017763, NP_060233; *NRAS*: NM_002524, NP_002515; *MAP2K1*: NM_002755, NP_002746; *TP53*: NM_000546, NP_000537; *KRAS*: NM_033360, NP_203524; *CDH1*: NM_004360, NP_004351; *FBXW7*: NM_033632, NP_361014.

#### *MUTYH*:c.1510_1517delinsCCAACAGCCA p.Thr504Profs*68

*MUTYH* variant c.1510_1517delinsCCAACAGCCA p.(Thr504Profs) was detected in *trans* (Suppl. Fig. 1b) with c.1187G > A; p.(Gly396Asp) in a male with more than 40 adenomas at the age of 56. In this instance, the subject did not present a family history of polyposis or CRC (Fig. 1a). Blood derived cDNA sequencing revealed an extension corresponding to 22 amino acids after the stop codon (p.Thr504Profs*68). The novel frameshift variation was located at the 3′-end of the coding sequence, altering the amino acid sequence of the whole proliferating cell nuclear antigen (PCNA) binding domain, which is essential for *MUTYH*’s activity during DNA replication^16^. Indeed, point mutations at the PCNA-binding domain have been proven to decrease the activity of the enzyme^17^. Somatic analysis of DNA from affected adenomas showed G > T changes at *KRAS* locus (Table 3, ID 89). Therefore, c.1510_1517delinsCCAACAGCCA p.Thr504Profs*68 was reclassified as class-5 (Suppl. Table 3).

#### *POLD1*:c.2007-5C > T

After cDNA analysis, no splicing alteration was detected in the c.2007-5 carrier. So it was reclassified as class-1.

#### *POLE*:c.6716C > T p.(Ala2239Val)

*POLE* variant c.6716C > T p.(Ala2239Val) was detected in homozygosis in both an affected and a healthy member, and it was absent in another affected member. Therefore, it was reclassified as class-2 variants.

### Co-occurrence of truncating variants at *POLE* and *NTHL1* loci

**Figure 2:**
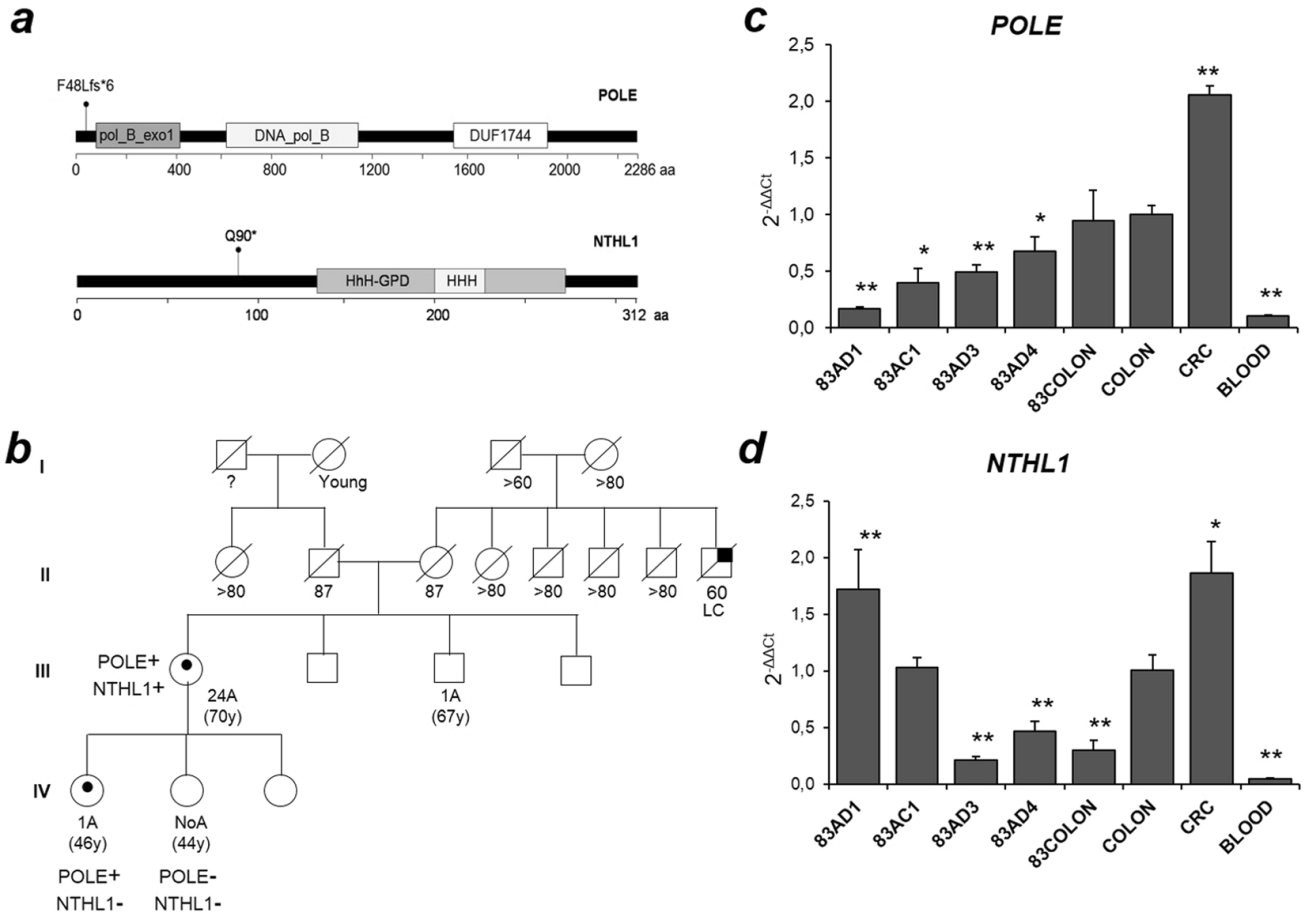
Identification of *POLE* and *NTHL1* double heterozygote. (**a**) POLE and NTHL1 protein domains and location of truncating variants detected in patient 83. (**b**) Pedigree chart; POLE+ = *POLE* variant carrier; POLE− = *POLE* wild type genotype; *NTHL1*+ = *NTHL1*variant carrier; *NTHL1*− = *NTHL1*wild type genotype; LC = lung cancer; A = adenoma. (**c**) *POLE* wild type expression in blood and tissue cDNA samples. (**d**) *NTHL1* wild type allele expression in blood and tissue cDNA samples. COLON pool sample has been used as a reference sample in both *NTHL1* and *POLE* assays. Significant ρ-values are indicated with *(ρ < 0.05) and **(ρ < 0.01). Each sample was analyzed in triplicates. AC = adenocarcinoma; AD = adenoma; CRC = colorectal cancer.

Two heterozygous truncating variants, *POLE*:c.141delG; p.Phe48Leufs*6 and *NTHL1*:c.268C > T; p.Gln90*, were detected in the same germline DNA (Fig. 2a). The patient was a woman with full-blown late AAP diagnosed at the age of 70 and without a previous family history of polyposis or CRC. Segregation analyses could only be achieved in two of her daughters, detecting *POLE*:c.141delG; p.Phe48Leufs*6 in one of them, who had been diagnosed of a low grade dysplastic tubular adenoma at the age of 46 (Fig. 2b).

In order to investigate the involvement of these variants in the adenoma and CRC formation, four adenomas (83AD1, 83AD3, 83AD4, 83AD5) and one adenocarcinoma (Haggit-0) (83AC1) from the proband were analyzed for somatic mutations (Table 3, ID 83). A total of 13 driver mutations was detected, two of which were indels, four C > A changes and seven were C > T changes. Eleven somatic mutations were located in genes involved in the early adenoma formation (*APC*, *RNF43* and *CTNNB1*), whereas two mutations were detected in genes involved in later stages (*ARID1A*, *NRAS*).

In addition, *NTHL1* and *POLE* specific wild type allele expression was checked at tissue level. Quantification analyses were performed in three adenomas (83AD1, 83AD3, 83AD4), one adenocarcinoma (83AC1) and healthy colon tissue from the proband (83COLON). *POLE* wild type allele expression in the carrier’s colon tissue (83COLON) did not show a significant change compared to the colon control pool (fold difference (FD) = 0.94 ± 0.27, ρ = 0.82), whereas *NTHL1* wild type allele expression in 83COLON did (FD = 0.30 ± 0.08, ρ = 0.004).

Both *POLE* and *NTHL1* transcripts were over-expressed in the CRC control pool (FD_POLE_ = 2.06 + 0.08, ρ = 0.003; FD_NTHL1_ = 1.87 + 0.28, ρ = 0.018). However, all adenomas and the adenocarcinoma tested from the carrier showed a decrease in the expression of *POLE* wild type allele (FD average = 0.46 ± 0.20, ρ < 0.001), whereas *NTHL1* wild type allele showed significant over-expression in one adenoma and one adenocarcinoma from the proband when compared to its healthy tissue (ρ = 0.0156 and ρ < 0.001, respectively) (Fig. 2c,d).

## Discussion

AAP is becoming one of the largest groups of patients attending our Genetic Counselling Unit. Genetic screening of *MUTYH* and *APC* is recommended in those cases with more than 10 adenomas^18^. The diagnostic approach in our hospital consists in the screening of the four most prevalent *MUTYH* mutations in Spanish population^19^, c.536A > G; p.(Tyr179Cys), c.1187G > A; p.(Gly396Asp), c.1227_1228dup; p.(Glu410Glyfs*43) and c.1437_1439delGGA; p.(Glu480del), and the subsequent screening of point mutations and copy number variations (CNVs) in the whole coding sequence of the *APC* gene in those cases with a higher polyp burden and high suspicion of inheritance pattern. However, by this approach we are only able to explain around 5% of the cases with more than 10 adenomas, or 7% when restricting the criteria to full-blown polyposis (more than 20 adenomas or 10 synchronous adenomas). In order to improve the diagnostic sensitivity and investigate the contribution of currently known high-penetrance adenomatous polyposis genes in our AAP population, we have conducted a genetic screening in an unexplained AAP cohort through a custom gene panel, including *APC*, *MUTYH*, *POLE*, *POLD1*, *NTHL1*, *AXIN2* and *MSH3* coding regions.

Our study cohort was made up of 158 subjects, 132 (83.5%) of which fulfilled the clinical criteria for full-blown polyposis (Table 1). There is only a very recent cross-sectional study analyzing the prevalence of pathogenic variants in genes associated with colorectal polyposis and/or CRC in a cohort of 3789 polyposis patients, of which 2979 presented between 10 and 100 adenomas. All patients underwent panel testing of at least 14 CRC predisposition genes, including *APC* and *MUTYH*. However, only *POLD1* and *POLE* were tested on a subset of the adenoma cohort, and the remaining adenomatous polyposis predisposition genes (*NTHL1*, *AXIN2* and *MSH3*) were not tested^20^. Therefore, this is the largest multiple polyposis cohort in which joint genetic screening for the full coding sequences of all AAP predisposition genes has been done so far.

Two *APC* truncating mutations were detected in two subjects diagnosed with late-onset full-blown polyposis and a family history initially suspicious of recessive inheritance (Fig. 1b). Both had been previously tested for *MUTYH* but not for *APC*, probably due to the lack of family information at the time of diagnosis, the late polyposis onset of the probands, and also the stricter clinical criteria for the recommendation of full *APC* mutational screening for AAP in our hospital.

In addition, the screening of the whole coding sequence of *MUTYH*, allowed the detection of two *MUTYH* non-recurrent mutations, c.739C > G p.(Arg247Gly) and c.1510_1517delinsCCAACAGCCA p.Thr504Profs*68, both in co-occurrence with the recurrent mutation c.1187G > A; p.(Gly396Asp), being able to explain the polyposis susceptibility in two more subjects (Fig. 1a).

*APC*/*MUTYH* mutational rates are low in AAP population^2^. Conventional genetic screening technologies imply the sequential analysis of each gene amplicon by amplicon, which makes these protocols costly in both time and money. Therefore, those laboratories without high sample volumes are forced to restrict their clinical criteria for AAP diagnosis in order to make the analysis cost-effective. NGS multigene panels reduce the time and cost of these genetic studies, increasing the cost-effectiveness and making the complete screening of samples feasible for small laboratories. The identification of pathogenic mutations in *APC* and *MUTYH* with an NGS panel in our study cohort is a clear example of underdiagnoses, supporting the necessity of parallel sequencing for *APC* and *MUTYH* routine genetic screening in AAP patients.

None of the patients showed actionable mutations (class-4 and -5) in any of the genes associated with new polyposis syndromes (*MSH3*, *NTHL1*, *POLD1, POLE* or *AXIN2*). However, 10 uncertain significance (class-3) variants were detected in *AXIN2*, *NTHL1*, *POLD1* and *POLE* (Table 2). Three of these variants were located outside the proofreading domain of *POLE*/*POLD1*. Although there is no evidence of association between missense variants outside these regions and cancer susceptibility, there is a recent report that describes somatic driver mutations located outside the exonuclease domains, and suggesting that other domains may be responsible for proofreading^21^. Therefore, we decided to include these variants as class-3 variants for AAP predisposition.

Considering other previous studies that included multiple polyposis cohorts, the frequency of actionable mutations in all of these genes seems to be very low in tested populations, but not insignificant (around 1–2%). Just like it is not irrelevant the number of variants of uncertain significance that have been described in this and other works^9,10,12^ (1–2% per gene), and whose probable pathogenicity might be proven during the coming years. For this reason it is important to include all these genes in the routine analysis of AAP through NGS panels.

A remarkable finding of this work is the detection of a double heterozygous for two truncating variants, at exon two of *NTHL1* and exon two of *POLE* (Fig. 2a). The carrier was a woman with late full-blown polyposis, who was also diagnosed with endometrial hyperplasia and hypothyroidism (Fig. 2b).

*POLE* encodes the catalytic subunit of DNA polymerase epsilon, which is responsible for the replication of the leading DNA strand during the S phase; it is an essential protein and biallelic truncating mutations are not viable. POLE, together with POLD1, is the only nuclear polymerase with an intrinsic 3′–5′exonuclease proofreading activity capable of correcting mistakes made during DNA synthesis^22^. A few years ago, germline mutations located in the proofreading domain of *POLE* and *POLD1* were associated with adenomatous polyposis predisposition^4^, showing tumors with a very high mutational burden due to the lack of exonuclease but not polymerase activity. According to this, truncating variants at *POLE* would not be supposed to confer this genetic instability because they would lead to a complete inactivation of the enzyme without any polymerase-exonuclease imbalance. However, it is not clear to what extent the lack of one *POLE* allele can lead to cancer predisposition in some other way.

On the other hand, *NTHL1* encodes the DNA glycosylase NTHL1, which is involved in removing oxidative pyrimidine lesions through the base excision repair (BER) pathway. Resembling to *MUTYH*-associated polyposis, germline biallelic mutations at *NTHL1* have been recently associated with adenomatous polyposis predisposition^5^, leading to a deficiency in the repair of 5-hydroxycytosine and a consequent increase in the C > T somatic mutation rate. Like other glycosylases involved in the BER pathway, NTHL1’s repair activity can be completed by a short or long BER patch, mainly depending on the proliferative activity of the cell^23^. Thus, in high division rate tissues NTHL1 is coupled to the DNA synthesis and follows the long-patch BER pathway, dependent of PCNA and where POLE and POLD1 are the polymerases responsible for filling the gap after NTHL1’s action and strand cleavage^23^.

Therefore, it is plausible that *POLE* and *NTHL1* co-occurring truncating variants may have a synergistic effect leading to a polyposis predisposition in high division tissues such as the colon epithelium. To check this hypothesis, somatic mutation screening and RNA expression analyses were performed in adenomas and CRC from the carrier. The analysis of somatic mutations showed a tendency to C > T changes (Table 3), although no clear definition of the mutational signature could be done due to the low number of mutations. Furthermore, a quantitative analysis of *POLE* wild type allele showed a significant decrease of *POLE* expression in all tested carrier’s adenomas and adenocarcinoma (Fig. 2c), which is consistent with a replicative stress due to POLE haploinsufficiency. However and unlike *POLE*, *NTHL1* expression was increased in two of the carrier’s adenomas and adenocarcinoma tissues (Fig. 2d). This over-expression was probably triggered by a greater oxidative DNA damage in tissues with a high division rate, which has been shown to up-regulate BER glycosylases^24^. Wild type *NTHL1* transcript levels in the carrier’s affected tissues reached similar levels than non-carrier’s affected pool, which is not consistent with an *NTHL1* haploinsufficiency. Therefore, we discarded the involvement of the *NTHL1* monoallelic mutation c.268C > T p.(Gln90*) in the polyposis predisposition of this patient and we considered the possibility that *POLE*:c.141delG p.Phe48Leufs*6 could confer the adenoma predisposition by itself. Only 0.005% of *POLE* coding variants described in gnomAD are truncating (frameshift or nonsense); non homozygous have been described and all truncating variants show allelic frequencies lower than 1/10000. Moreover, there are two studies in the literature describing germline frameshift variants at *POLE*; c.5621_5622delGT was detected in a sporadic CRC patient with a diagnosis age of 26^25^, and c.1370_1371delAT p.Tyr457fs*9 was later detected in an AAP patient^10^. In addition, as it has been mentioned above, somatic driver mutations have been recently described in *POLE* and *POLD1* polymerase domains^21^.

Furthermore, the presence of both variants was checked in two of the proband’s daughters; one harbored the *POLE* variant, but not the *NTHL1* one, and the other did not harbor any of the variants (Fig. 2b). The *POLE* carrier showed a dysplastic tubular adenoma at the age of 46. Despite the large size of the family, no other relatives had been diagnosed of cancer or polyposis, which would support the hypothesis of a digenic or oligogenic inheritance with other undetected variants or, a *de novo POLE* mutation in the proband with a dominant effect that is not yet possible to observe due to the young age of the daughter harboring the variant (Fig. 2b).

To our knowledge, this is the first report describing the probable association of a truncating germline *POLE* variant with the predisposition of AAP by a haploinsufficiency mechanism in adenomatous and colorectal tumor tissues of the carrier. This result highlights not only the genetic heterogeneity and complexity of the syndrome, but also the potential of NGS gene panels for the detection and diagnosis of new inheritance forms of complex diseases. Further efforts should be done for the study and characterization of germline *POLE* truncating variants in other AAP populations, as well as their co-occurrence with variants in other associated genes.

In accordance with other AAP studies, our results showed a major number of subjects without detection of variants in any of the genes tested. It can be thought the involvement of other unknown predisposition genes in the susceptibility of AAP and the convenience of wider genetic analysis, such as exome sequencing, for the elucidation of new AAP predisposition genes in this unexplained group. However, despite most of the new AAP associated genes have been discovered by exome sequencing approaches, there are other works without such successful results^26,27^. The failure of detecting new predisposition genes in AAP is probably due to the high clinical and genetic heterogeneity of the condition, as well as the low prevalence of pathogenic mutations in the already associated genes. Probably, polygenic inheritance models in which the susceptibility is explained by the accumulation of multiple low penetrance alleles^28^, and lifestyle risk factors such as smoking, alcohol, body mass index, diet and physical activity^29^ play a major role in the unexplained AAP.

In our cohort, all patients with pathogenic mutation detection presented full blown AAP (more than 20 adenomas) and an average diagnosis age lower than the general study cohort (57.2). Other works analyzing AAP cohorts, such as Grover^30^ who analyzed *MUTYH* and *APC* in 4223 patients with 10 to 100 adenomas, or Stanich^20^ who also analyze *POLD1* and *POLE* and other CRC genes in 2979 patients with 10 to 100 adenomas, showed similar clinical results. These results suggest that the low mutation detection rate in AAP is partially because of the lack of strict clinical criteria for the selection of patients with high probability to detect pathogenic mutation in any known predisposition gene. Therefore, redefinition of stronger clinical criteria and the use of panel gene testing are necessary for the improvement of genetic testing in AAP.

This work is a translational study aiming to analyze the contribution of known and new described adenomatous polyposis predisposition genes and the suitability of their genetic testing in a hospital based cohort that have been referred to the genetic counseling unit. The results shown above lead to the following conclusions:

1. The contribution of new predisposition genes is much smaller than that of the known genes *APC* and *MUTYH*. Related pathogenic mutations have not been detected in any gene. However, uncertain significance variants have been detected in all genes but *MSH3*. Since they are recently described genes, the identification of potential pathogenic variants and further clarification of their pathogenicity is important for the definition of the syndrome.
2. Somatic genetic screening of affected tissues allows the detection of certain mutational signatures associated with DNA repair deficiency, helping the classification of the variants.
3. The expected low mutation detection rate in AAP study cohort point to the necessity of stronger clinical criteria for the improvement of the diagnostic sensitivity in AAP genetic testing.
4. Although the number of *POLE/POLD1* truncating mutations detected in AAP cases is very limited, due to the genetic heterogeneity of the disease, the relevance of these genes and the decreased levels of carrier’s tumor and adenoma samples shown in this work, special attention should be paid to those *POLE/POLD1* truncating variants in order to determine their pathogenicity.
5. Summarizing, this work highlights the need of multigene panel testing in highly genetic heterogeneous syndromes such as AAP, not only to increase the cost-effectiveness and the diagnostic sensitivity of the analysis, but also to better detect other potentially pathogenic variants or other inherited forms of the disease that would not be detected by other gene directed approaches.

## Patients and Methods

### Patients

The inclusion criterion was the detection of between 10 and 100 adenomas in the colon and/or rectum. All the samples had been screened, at least, for the most frequent *MUTYH* (NM_0012222.2) mutations in the Spanish population^19^: c.536A > G; p.(Tyr179Cys), c.1187G > A; p.(Gly396Asp), c.1227_1228dup; p.(Glu410Glyfs*43) and c.1437_1439delGGA; p.(Glu480del), by HRM and/or Sanger sequencing. The presence of biallelic *MUTYH* mutations was considered as an exclusion criterion, but not the presence of monoallelic *MUTYH* mutations. *APC* screening was not an inclusion criterion, but the previous detection of a pathogenic *APC* mutation was considered as an exclusion criterion.

A total of 158 unrelated AAP cases was included in the study (Suppl. Table 1), comprising 123 subjects from Hospital Clínico San Carlos (Madrid) and 35 from Hospital 12 de Octubre (Madrid).

Ethical approval was obtained from Hospital Clínico San Carlos’ Ethical Research Committee (approval number: C.I.-14/241-E_BS). A written informed consent was obtained from each participant. Methods were compliant with the relevant guidelines and regulations.

### Sample extraction

Peripheral-blood DNA/RNA extraction was performed with the MagNA Pure Compact extractor (Roche), according to the manufacturer’s protocol. Tissue DNA/RNA (from normal epithelium, adenoma or tumor sections) was obtained from formalin-fixed paraffin-embedded (FFPE) tissues with a purity greater than 80% as determined by an experienced pathologist. DNA extractions were performed with the QIAamp DNA FFPE Tissue kit (Qiagen N.V.) and RNA extractions with the RNAeasy FFPE kit (Qiagen N.V.). SuperScript First-Strand Synthesis System for RT-PCR (Thermo Fisher Scientific) was used to synthesize cDNA, either from blood or tissue RNA, using random hexamers and oligo-dT, according to the manufacturer’s instructions.

### Germline DNA screening

Germline DNAs were screened for *APC*, *MUTYH*, *POLE*, *POLD1*, *NTHL1*, *MSH3* and *AXIN2* variants in the coding regions and intron-exon boundaries through an NGS Haloplex custom panel (Agilent Technologies). Enriched libraries were obtained according to the manufacturer’s protocol, and subsequently sequenced on a MiSeq System (Illumina, Inc.). Data analysis and variant calling were achieved with the SureCall software (Agilent Technologies) following the recommended pipeline for Haloplex libraries. Low covered regions (read depth <50) were analyzed by HRM. CNVs analyses at *APC* and *MUTYH* loci were performed by multiplex ligation and probe amplification (MLPA) (SALSA^®^ MLPA^®^probemix P043 and P378, MRC-Holland).

All rare (novel or minor allele frequency (MAF) <0.01 according to the gnomAD^31^ and 1000 genomes project^32^ databases), deleterious or possible deleterious variants (according to protein and/or splicing alteration prediction tools) were selected for validation by Sanger sequencing. MaxEnt, and human splicing finder (HSF)^33^ were used to predict splicing alterations, while SIFT^34^, Polyphen2^35^ and MutationTaster^36^ predicted protein damage.

### Classification of variants

Variants were classified in five different pathogenicity classes (class-5 or pathogenic, class-4 of likely pathogenic, class-3 or uncertain significance, class-2 or likely benign and class-1 or benign) according to the public mutational databases (InSight^37^ LOVD^38^, UMD^39^, ClinVar^40^) and supporting pathogenic and/or benign evidences, in accordance to the ACMG-SHERLOC criteria^41,42^.

### Characterization of variants

#### Segregation analyses

Whenever possible, segregation analyses of candidate variants were performed on DNA from available family members by Sanger sequencing (oligonucleotides shown in Suppl. Table 2).

#### Splicing analyses

Blood derived cDNAs from patients harboring variants either with a positive splicing alteration prediction or located between five nucleotides from the intron-exon boundaries, were subjected to transcript analyses by Sanger sequencing (oligonucleotides shown in Suppl. Table 2).

#### Adenoma/tumor DNA screening

Adenoma and tumor DNAs were screened for somatic mutations using the commercial amplicon-based TruSight Tumor 26 panel (TST26) (Illumina, Inc.), which includes 174 relevant regions located in 26 genes involved in solid tumors. Sequencing was performed on a MiSeq System (Illumina, Inc.). Data analysis and variant calling were performed through the plug-in specific Amplicon DS workflow for the MiSeq Reporter Software tool (Illumina, Inc.). PASS filter variants, according to the default settings, were first selected. Variants that failed to pass default filters, but that were detected in both pools (i.e. showing a low coverage depth or strand bias) were manually inspected using the Integrative Genome Viewer (IGV) browser^43^. Known germline polymorphisms, variants detected in the majority of samples tested, variants previously detected in the patient’s germline DNA or present in all tested samples from the same subject were discarded.

By single-molecule molecular inversion probe (smMIP) sequencing, two adenomas (83AD1 and 83AD4) and one adenocarcinoma (83AC1) were also investigated for the occurrence of somatic mutations in the open reading frames and hotspot regions of 57 genes involved in CRC development, as described previously^44^.

In order to characterize the variant *MUTYH*:c.1510_1517delinsCCAACAGCCA p.(Thr504Profs*68), three adenomas of patient 89 were analyzed for *KRAS* somatic mutations at codons 12 and 13 by the Therascreen *KRAS* RGQ PCR Kit (Qiagen N.V.), according to the manufacturer’s protocol.

#### cDNA expression analyses

*NTHL1* and *POLE* wild type allele expression levels were evaluated in three adenomas (83AD1, 83AD3 and 83AD4), one adenocarcinoma (83AC1) and one healthy colon tissue (83COLON) from patient 83 and compared to healthy colon and colorectal cancer control pools (COLON and CRC). The healthy colon pool was made up of eight FFPE colon tissue-derived RNA samples from unrelated healthy subjects, while the colon cancer pool was made up of eight FFPE CRC tissue-derived RNA samples from unrelated patients. All samples were previously treated with RNase-free recombinant DNase I (Roche) and the lack of germline DNA traces was checked by specific germline and cDNA amplification (Suppl. Fig. 2, primer sequences on Suppl. Table 2).

For each of the genes to be tested, primer pairs were designed for the specific detection of the wild type allele, and *PSMB4* was used as an endogenous gene (Suppl. Table 2). For each sample, 5 ng/μL of RNA was amplified in triplicates with the KAPA SYBR® FAST Universal Kit (Roche) in a Light Cycler® 96 (Roche), according to the manufacturer’s instructions.

### Reclassification of variants

Reclassification of variants was achieved according to the supporting pathogenic and/or benign evidences, in accordance to the ACMG-SHERLOC criteria^41,42^

## Supplementary information

Supplementary information

## Data Availability

The datasets generated during and/or analyzed during the current study are available from the corresponding author on reasonable request.
