# Supplementary material for: Contribution of New Adenomatous Polyposis Predisposition Genes in an Unexplained Attenuated Spanish Cohort by Multigene Panel Testing
Source: Sci Rep. 2019 Jul 8;9:9814. doi: 10.1038/s41598-019-46403-5 (PMC6614360; doi:10.1038/s41598-019-46403-5)
Supplement: Supplementary file 1 — Supplementary information [file 41598_2019_46403_MOESM1_ESM.pdf]

SUPPLEMENTARY INFORMATION

CONTRIBUTION OF NEW ADENOMATOUS POLYPOSIS PREDISPOSITION GENES IN AN UNEXPLAINED ATTENUATED SPANISH COHORT BY MULTIGENE PANEL TESTING

Víctor Lorca[1], Daniel Rueda[2], Lorena Martín-Morales[1], María Jesús Fernández-Aceñero[3], Judith Grolleman[4], Carmen Poves[5], Patricia Llovet[1],Sandra Tapial[2], Vanesa García-Barberán[1], Julián Sanz[3], Pedro Pérez-Segura[6], Richarda M. de Voer[4], Eduardo Díaz-Rubio[6], Miguel de la Hoya[1], Trinidad Caldés[1], Pilar Garre[1*].

[1]Laboratorio de Oncología Molecular, Hospital Clínico San Carlos; IdISSC, Madrid, Spain, CIBERONC. [2]Laboratorio de Cáncer Hereditario, Servicio de Bioquímica, i+12, Hospital 12 de Octubre, Madrid, Spain. [3]Servicio de Anatomía Patológica, Hospital Clínico San Carlos, Madrid, Spain. [4]Department of Human Genetics, Radboud University Medical Center, Nijmegen, The Netherlands. [5]Servicio de Aparato Digestivo, Hospital Clínico San Carlos, Madrid, Spain. [6]Servicio de Oncología Médica. Hospital Clínico San Carlos, Madrid, Spain, CIBERONC.

*Correspondingauthor: **Pilar Garre**, Laboratorio de Oncología Molecular, Hospital Clínico San Carlos. c/ Profesor Martín Lagos s/n, 28040 Madrid, Spain. Tel.: 00(34)913303348. Fax: 00(34)913303544 E-mail: pilar_garre@hotmail.com; pilar.garre@salud.madrid.org

Supplementary table 1. Clinical characteristics of participants.

ID	Origin[a]	Gender[b]	Criteria[c]	Age of diagnosis	N of polyps	N of colonoscopies	Type of polyposis[d]	Colectomy	CRC	Inheritance[e]	*APC* screening[f]	*MUTYH*screening[g]
1	HCSC	M	>20A	72	42	8	A	NO	NO	I	NO	REC
2	HCSC	F	>20A	71	20	8	A	NO	NO	R	NO	REC
3	HCSC	F	>20A	57	96	14	A	NO	NO	R	NO	REC
4	HCSC	M	>10A	57	19	6	A	NO	YES	D	NO	REC
5	HCSC	M	>10SA	58	14	7	A	YES	YES	D	NO	REC
6	HCSC	M	>20A	72	41	7	A	YES	YES	I	NO	REC
7	HCSC	M	>20A	64	20	5	M	NO	NO	I	NO	REC
8	HCSC	M	>20A	54	51	9	M	NO	NO	R	NO	REC
9	HCSC	F	>20A	62	61	7	A	NO	NO	D	NO	REC
10	HCSC	M	>20A	64	21	4	M	YES	NO	R	FULL	REC
11	HCSC	M	>20A	42	21	15	M	NO	YES	R	FULL	REC
12	HCSC	M	>20A	62	28	4	M	NO	NO	I	FULL	REC
13	HCSC	M	>20A	43	26	4	A	YES	YES	R	FULL	REC
14	HCSC	F	>10SA	57	20	6	M	YES	YES	I	NO	REC
15	HCSC	M	>20A	63	52	8	M	YES	YES	I	NO	REC
16	HCSC	M	>20A	67	29	9	A	NO	NO	R	NO	REC
17	HCSC	F	>20A	71	51	3	M	NO	NO	R	FULL	REC
18	HCSC	M	>20A	68	34	5	A	NO	NO	R	NO	REC
19	HCSC	M	>20A	69	31	4	M	YES	NO	I	FULL	REC

ID	Origin[a]	Gender[b]	Criteria[c]	Age of diagnosis	N of polyps	N of colonoscopies	Type of polyposis[d]	Colectomy	CRC	Inheritance[e]	*APC* screening[f]	*MUTYH*screening[g]
20	HCSC	M	>20A	75	31	4	A	YES	YES	I	FULL	REC
21	HCSC	M	>20A	61	26	10	A	NO	NO	D	NO	REC
22	HCSC	M	>20A	64	60	9	A	NO	NO	R	NO	REC
23	HCSC	F	>20A	72	26	9	M	NO	NO	I	NO	REC
24	HCSC	M	>20A	52	41	11	M	YES	NO	R	NO	REC
25	HCSC	M	>20A	74	30	4	A	NO	NO	I	NO	REC
26	HCSC	M	>20A	60	36	9	A	NO	NO	I	NO	REC
27	HCSC	M	>20A	49	35	10	M	YES	NO	R	NO	REC
28	HCSC	F	>20A	68	21	5	M	NO	NO	I	NO	REC
29	HCSC	M	>20A	74	49	10	M	YES	YES	I	NO	REC
31	HCSC	F	>10A	56	18	3	A	NO	NO	R	NO	REC
32	HCSC	F	>10SA	57	26	7	M	NO	YES	R	NO	REC
34	HCSC	M	>20A	74	24	3	M	NO	NO	I	NO	REC
35	HCSC	M	>20A	37	21	7	A	NO	NO	R	NO	REC
36	HCSC	M	>20A	55	30	13	M	YES	YES	R	NO	REC
37	HCSC	M	>20A	65	33	10	A	YES	YES	R	NO	REC
38	HCSC	F	>20A	47	34	10	M	NO	NO	R	NO	REC
39	HCSC	M	>20A	72	45	4	A	NO	YES	R	NO	REC
40	HCSC	F	>20A	57	25	4	M	NO	NO	I	NO	REC
41	HCSC	M	>20A	66	21	8	A	NO	NO	R	NO	REC
42	HCSC	M	>20A	77	31	2	A	NO	NO	I	NO	REC

ID	Origin[a]	Gender[b]	Criteria[c]	Age of diagnosis	N of polyps	N of colonoscopies	Type of polyposis[d]	Colectomy	CRC	Inheritance[e]	*APC* screening[f]	*MUTYH*screening[g]
43	HCSC	M	>10SA	61	20	5	M	NO	NO	R	NO	REC
44	HCSC	M	>10A	67	17	7	M	NO	NO	I	NO	REC
47	HCSC	M	>20A	36	41	10	A	YES	YES	I	NO	REC
48	HCSC	F	>20A	66	43	11	A	YES	NO	R	FULL	REC
49	HCSC	M	>10A	68	15	4	A	NO	NO	I	NO	REC
50	HCSC	F	>20A	63	32	4	M	NO	NO	R	NO	REC
51	HCSC	M	>10A	61	14	6	M	NO	NO	I	NO	REC
52	HCSC	F	>10SA	55	15	1	A	YES	NO	D	FULL	REC
53	HCSC	M	>10SA	60	17	6	A	NO	NO	R	NO	REC
54	HCSC	M	>20A	62	23	6	M	NO	NO	I	NO	REC
55	HCSC	F	>10A	72	14	10	M	NO	YES	D	FULL	REC
56	HCSC	M	>10SA	74	18	3	A	NO	NO	I	NO	REC
57	HCSC	M	>20A	72	24	5	A	YES	NO	I	NO	REC
58	HCSC	M	>20A	75	20	9	M	NO	YES	I	NO	REC
59	HCSC	M	>20A	73	26	6	A	YES	YES	D	NO	REC
60	HCSC	M	>10SA	66	19	4	A	NO	NO	I	NO	REC
61	HCSC	M	>10A	54	15	7	A	NO	YES	I	NO	REC
62	HCSC	M	>20A	67	21	9	M	NO	NO	I	NO	REC
63	HCSC	M	>10A	64	13	4	M	NO	NO	I	NO	REC
64	HCSC	M	>20A	64	37	8	M	NO	NO	I	NO	REC
65	HCSC	M	>10A	70	16	9	M	NO	YES	R	NO	REC

ID	Origin[a]	Gender[b]	Criteria[c]	Age of diagnosis	N of polyps	N of colonoscopies	Type of polyposis[d]	Colectomy	CRC	Inheritance[e]	*APC* screening[f]	*MUTYH*screening[g]
66	HCSC	F	>20A	55	31	2	A	YES	YES	I	NO	REC
67	HCSC	M	>20A	51	100	4	A	YES	NO	I	FULL	REC
68	HCSC	M	>10A	60	17	3	M	NO	NO	I	NO	REC
69	HCSC	F	>20A	70	23	3	M	NO	NO	I	NO	REC
70	HCSC	M	>10SA	66	16	4	A	NO	NO	I	NO	REC
71	HCSC	M	>20A	33	21	3	A	YES	NO	R	NO	REC
72	HCSC	M	>10SA	63	14	2	M	YES	YES	I	NO	REC
73	HCSC	M	>10A	56	14	6	M	NO	NO	I	NO	REC
74	HCSC	F	>10A	70	11	4	A	NO	NO	I	NO	REC
75	HCSC	M	>20A	64	31	1	M	NO	YES	I	NO	REC
76	HCSC	M	>10A	68	15	8	A	NO	NO	D	NO	REC
77	HCSC	M	>10A	62	18	4	A	NO	NO	D	NO	REC
78	HCSC	M	>10SA	47	44	10	M	NO	NO	I	NO	REC
79	HCSC	M	>10A	67	15	4	A	NO	NO	R	NO	REC
80	HCSC	M	>10SA	61	18	2	M	NO	NO	I	NO	REC
81	HCSC	F	>20A	55	32	7	A	NO	NO	I	NO	REC
82	HCSC	M	>10SA	80	18	4	A	NO	NO	I	NO	REC
83	HCSC	F	>20A	74	24	5	A	NO	NO	I	NO	REC
84	HCSC	M	>10A	68	12	3	M	NO	NO	I	NO	REC
85	HCSC	M	>20A	62	21	4	M	NO	NO	R	NO	REC
87	HCSC	M	>20A	67	29	6	A	NO	NO	D	NO	REC

ID	Origin[a]	Gender[b]	Criteria[c]	Age of diagnosis	N of polyps	N of colonoscopies	Type of polyposis[d]	Colectomy	CRC	Inheritance[e]	*APC* screening[f]	*MUTYH*screening[g]
88	HCSC	M	>20A	70	22	6	M	NO	NO	R	NO	REC
89	HCSC	M	>20A	56	40	2	A	NO	NO	I	NO	REC
90	HCSC	M	>20A	76	22	3	M	YES	NO	I	NO	REC
91	HCSC	M	>10A	57	12	5	A	NO	NO	D	NO	REC
92	HCSC	M	>10SA	72	19	4	A	NO	NO	I	NO	REC
94	HCSC	M	>10SA	73	21	5	M	NO	NO	I	NO	REC
95	HCSC	M	>10SA	74	20	4	A	NO	YES	I	NO	REC
96	HCSC	F	>20A	70	39	6	M	YES	YES	R	NO	REC
97	HCSC	F	>20A	71	24	10	M	NO	NO	I	NO	REC
98	HCSC	M	>10A	64	10	7	A	NO	NO	R	NO	REC
99	HCSC	M	>10SA	79	18	3	M	NO	NO	I	NO	REC
100	HCSC	M	>10A	77	10	9	A	NO	NO	R	NO	REC
101	HCSC	M	>10A	55	16	7	M	NO	NO	I	NO	REC
102	HCSC	F	>10SA	68	10	2	A	YES	YES	R	NO	REC
103	HCSC	M	>10SA	65	24	5	M	NO	NO	D	NO	REC
104	HCSC	F	>10SA	63	21	5	M	NO	YES	I	NO	REC
105	HCSC	F	>20A	67	20	3	A	NO	NO	D	NO	REC
106	HCSC	M	>20A	51	100	10	M	YES	YES	R	NO	REC
107	HCSC	M	>10SA	80	12	5	A	NO	NO	D	NO	REC
108	HCSC	F	>10SA	64	22	6	M	NO	NO	I	NO	REC
109	HCSC	M	>20A	47	20	6	A	NO	NO	D	NO	REC

ID	Origin[a]	Gender[b]	Criteria[c]	Age of diagnosis	N of polyps	N of colonoscopies	Type of polyposis[d]	Colectomy	CRC	Inheritance[e]	*APC* screening[f]	*MUTYH*screening[g]
110	HCSC	F	>20A	63	30	5	A	NO	NO	I	NO	REC
111	HCSC	F	>10SA	56	19	5	M	NO	NO	R	NO	REC
112	HCSC	F	>10A	61	15	8	A	YES	NO	R	NO	REC
113	HCSC	M	>10A	60	10	3	A	NO	NO	D	NO	REC
114	HCSC	F	>20A	55	35	10	M	NO	NO	I	NO	REC
115	HCSC	M	>10A	55	16	3	M	NO	NO	R	NO	REC
116	HCSC	F	>10SA	47	24	5	M	YES	YES	D	NO	REC
117	HCSC	M	>10SA	62	21	3	M	-	NO	R	NO	REC
118	HCSC	F	>10A	57	12	3	A	-	NO	R	NO	REC
119	HCSC	M	>10A	64	13	5	M	-	NO	R	NO	REC
121	HCSC	M	>20A	58	65	8	M	-	NO	R	NO	REC
122	HCSC	F	>10A	50	17	7	M	-	NO	R	NO	REC
123	HCSC	M	>10SA	80	11	2	A	-	NO	I	NO	REC
125	H12O	M	>20A	47	45	-	A	-	NO	I	FULL	REC
126	H12O	F	>20A	40	55	-	A	-	YES	D	FULL	FULL
127	H12O	M	>20A	65	45	-	A	-	NO	I	FULL	REC
128	H12O	M	>20A	64	51	16	A	YES	YES	I	NO	REC
129	H12O	M	>20A	61	51	8	A	YES	NO	D	NO	REC
130	H12O	M	>20A	72	41	-	A	-	NO	I	NO	REC
131	H12O	M	>20A	79	51	6	A	NO	NO	I	NO	REC
132	H12O	M	>10SA	68	42	10	M	NO	NO	I	NO	REC

ID	Origin[a]	Gender[b]	Criteria[c]	Age of diagnosis	N of polyps	N of colonoscopies	Type of polyposis[d]	Colectomy	CRC	Inheritance[e]	*APC* screening[f]	*MUTYH*screening[g]
133	H12O	F	>20A	54	43	6	M	NO	NO	D	NO	REC
134	H12O	M	>20A	78	51	11	A	NO	NO	D	NO	REC
135	H12O	M	>20A	58	51	13	M	NO	NO	I	NO	REC
136	H12O	M	>20A	66	48	-	A	-	NO	I	NO	REC
137	H12O	M	>20A	76	51	1	A	YES	YES	I	FULL	FULL
138	H12O	M	>20A	68	38	-	M	-	NO	I	NO	REC
139	H12O	M	>20A	72	81	4	A	YES	YES	D	FULL	FULL
140	H12O	M	>20A	76	31	8	A	NO	NO	I	NO	REC
141	H12O	M	>20A	61	41	-	A	-	NO	D	NO	REC
142	H12O	F	>20A	64	32	-	A	-	NO	R	NO	REC
143	H12O	M	>20A	60	61	-	M	-	NO	D	FULL	REC
144	H12O	M	>20A	69	81	-	A	-	YES	I	FULL	REC
145	H12O	F	>20A	52	50	-	A	-	NO	I	NO	REC
146	H12O	M	>20A	50	31	5	A	YES	NO	D	NO	REC
147	H12O	F	>20A	51	36	5	A	-	NO	D	NO	REC
148	HCSC	F	>10SA	73	17	3	M	-	NO	D	NO	REC
149	HCSC	M	>20A	77	25	4	M	-	NO	D	NO	REC
150	HCSC	M	>20A	50	20	5	A	-	NO	R	NO	REC
151	H12O	M	>20A	48	31	-	A	YES	NO	D	NO	REC
152	H12O	M	>20A	68	40	-	A	-	NO	D	NO	REC
153	H12O	M	>20A	70	51	2	A	YES	YES	R	FULL	REC

ID	Origin[a]	Gender[b]	Criteria[c]	Age of diagnosis	N of polyps	N of colonoscopies	Type of polyposis[d]	Colectomy	CRC	Inheritance[e]	*APC* screening[f]	*MUTYH*screening[g]
154	H12O	M	>20A	69	76	-	A	-	YES	D	FULL	REC
155	H12O	M	>20A	67	34	-	A	-	NO	R	NO	REC
156	H12O	M	>20A	69	30	-	A	-	YES	I	NO	REC
157	H12O	M	>20A	68	51	12	A	NO	NO	D	FULL	REC
158	H12O	M	>20A	54	42	-	A	-	YES	R	FULL	FULL
159	H12O	F	>20A	53	31	7	A	NO	NO	I	NO	REC
160	H12O	M	>20A	64	31	-	A	YES	NO	I	FULL	FULL
161	H12O	F	>20A	54	31	5	A	YES	NO	I	NO	REC
162	HCSC	M	>20A	52	31	7	M	NO	NO	D	NO	REC
163	HCSC	F	>20A	54	51	8	M	NO	NO	R	NO	REC
164	HCSC	M	>20A	79	37	6	M	YES	YES	D	NO	REC
165	HCSC	M	>20A	54	38	13	M	NO	NO	I	NO	REC
167	H12O	M	>20A	60	49	11	M	YES	NO	D	NO	REC

[a]Origin: HCSC= Hospital Clínico San Carlos, H12O: Hospital 12 de Octubre; [b]Gender: M= Male, F= female; [c]Criteria: >10A= more than 10 adenomas, >10SA= more than 10 synchronic adenomas, >20A= more than 20 adenomas; [d]Type of polyposis: A= adenomatous, M= mixed; [e]Inheritance: I= isolated, D= dominant, R= recessive.[f]*APC* Testing: FULL= Previous screening of whole *APC*coding sequence by sequencing and large rearrangements by MLPA, NO=Noprevious *APC* Screening; [g]*MUTYH*Testing:FULL= Previous screening of whole *MUTYH*coding sequence by sequencing, REC= Previous screening of *MUTYH* prevalent mutations. Empty cells indicate that there are not any screening data.

Supplementary table 2. List of oligonucleotides.

GENE	EXON	PRIMER[a]	SEQUENCE (5'->3')	STUDY[b]
APC	ex3	F	AAGGTGCGTGCTTTGAGAGT	VAL
		R	AAGCTGTACTTGGATCTACACACC	
APC	ex4	F	TTACCCTGACCCAAGTGGAC	VAL
		R	CTGGAGTACACAAGGCAATGTT	
APC	ex16	F	TGTTACTGCATACACATTGTGACC	VAL
		R	CCATGTCCCATAATGCTTCC	
APC	ex16	F	GGAAGGCAAAGTCCTTCACA	VAL
		R	CCCGTGACCTGTATGGAGAA	
APC	ex16	F	GAGTCTGCCTCCAAAGGACT	VAL
		R	GGGACCTAGTGGGAGAAGC	
APC	ex16	F	AGAATCCAGTGGAACCCAAA	VAL
		R	TGGCTTCCAGAACAAAAACC	
APC	ex10	F	GCAGCACTCCACAACATCAT	VAL
		R	CACATTTGCTTTGAAACATGC	
AXIN2	ex8	F	CCCAGTTTCTTTCCTTCTGTTTT	VAL
		R	GCCTCAACCTAGGACCCTTC	
AXIN2	ex2	F	AGCAGCAGCTTCCGTGAG	VAL
		R	CTTGATCGCCCAATAAGGAG	
MUTYH	ex13	F	GGCAGTGGCATGAGTAACAAG	VAL
		R	CTTGCGCTGAAGCTGCTCT	
MUTYH	ex9	F	CAGCCCAGGCTAACTCTTTG	VAL
		R	AGAGCTCCTTTGCAGACACC	
MUTYH	ex8	F	GGGTAGGAACCCAGGAGTCT	VAL
		R	AGAGGGGCCAAAGAGTTAGC	
MUTYH	ex15	F	GGACATGAAGTTAAGGGCAGA	VAL
		R	AGTGAAGCCTGGAGTGGAGA	
POLD1[4]	ex17	F	AGTGTGCTTTCCCCGTGTT	VAL
		R	GAGTGCCCACCTGTGAGAT	
POLD1	ex5	F	TCTGATCATCCCTCCCACAC	VAL
		R	AAGCTGGGACCAGCCAAT	
POLD1	ex12	F	GTGTGTCCCTGTCCTTGGAA	VAL
		R	GTTGGGGTGAGAGGTCAGG	
POLE	ex31	F	GCATTCCCATCTCACCACTT	VAL
		R	CTCCCCTTGGATCAAGGTCT	
POLE	ex48	F	AGCCTGTGAAGAAGCAGCAG	VAL
		R	CACTCAGAGAGGAGGCCAAG	
POLE	ex2	F	AAAAAGAAGCAGCAGCAGGT	VAL
		R	CATATTCCTGGGTGGGAGAA	
AXIN2	ex6 - ex9	F	CATAGTGCCCAAAGCACAAA	SPL
		R	CATCCTCCCAGATCTCCTCA	
MUTYH	ex11 - ex16	F	GTCCTGACGTGGAGGAGTGT	SPL
		R	AATGGGGGCTTTCAGAGGTG	
POLD1	ex15 - ex20	F	TGTGTTACACCACGCTCCTT	SPL
		R	AGTGAGGCAGTGACCAGGTT	
NTHL1	ex1	F	GGAGCTTGCTGGGAGTTGTA	HRM
		R	CAGCCTGCAGCCCCTATC	
NTHL1	ex2	F	ACCCCTACCCTACCTTCACC	HRM
		R	GGACCTTGCTAAGATGGGGG	
NTHL1	ex3	F	GGTCCCTGTCACTGCACAA	HRM
		R	TTGACCCTCACTTCCTGCAC	
NTHL1	ex4	F	TGAACCCACCCCTGTCTTTC	HRM
		R	GAATCCCAAGAGCAGCCAGT	
NTHL1	ex5	F	GGCTAGGCTGGTGGAGTGT	HRM
		R	GGGGTGAGCTCTTCTCCCTA	
NTHL1	ex6	F	GAGTGGCTGCCTAGGTATGA	HRM
		R	TCCTGAAGCGTAAAGCCACTT	
PALB2	ex2-ex3	F	AAATTAGCATTCTTGAAAAGGGAAT	DNAse
		R	GAGTGTTTTAGCTGCGGTGA	
NTHL1	ex2	F	GTGGCCTATGAGGGCTCGGA	qPCR

GENE	EXON	PRIMER[a]	SEQUENCE (5'->3')	STUDY[b]
		R	AGCTGTTGCTGCCAGTCCTG	
POLE	ex2-ex3	F	CGGATAAGATGGATTTGCGGT	qPCR
		R	AATCTCGGTAGGATGCATGTT	
PSMB4	ex5-ex6	F	CTCTGCTGCGAGAAGTTCTG	qPCR
		R	GGCGATTTGAAACCGGTTGT	

[a]PRIMER: F= Forward, R= Reverse; [b]STUDY: VAL= Variant validation; SPL= Splicing analyses; HRM= High Resolution Melting screening; DNAse: Analysis used to check genomic DNA contamination after DNAse treatment; qPCR: Transcript expression analyses by qPCR. Reference sequences: *APC*: NM_000038; *AXIN2*: NM_004655; *MUTYH*: NM_001128425; *POLD1*: NM_001256849; *POLE*: NM_006231;*NTHL1*: NM_002528; *PALB2:* NM_024675; *PSMB4: NM_002796.*

Supplementary table 3A. Reclassification of class 3 variants.

GENE	ID	HGVS designation	Segregation		Splicing Analyses		G>T somatic changes[d]	CLASS[e]
			Affected[a]	Healthy[b]	Prediction[c]	cDNA		
APC	***34***	c.1966C>G p.(Leu656Val)			NP			3
	133	c.7399C>A p.(Pro2467Thr)			NP			3
	136	c.8501A>C p.(His2834Pro)			NP			3
	139	c.1240C>G p.(Arg414Cys)	2/2	0/1	NP			3
AXIN2	***55***	c.2141G>A p.(Arg714Gln)	2/2		NP(+/-5)	wt		3
	79	c.203G>A p.(Arg68Gln)			NP			3
MUTYH	***35***	c.739C>G p.(Arg247Gly)	2/2		NP		4/6	4
	61	c.667A>G p.(Ile223Val)			NP		0/3	3
	89	c.1510_1517delinsCCAACAGCCA p.Thr504Profs*68			DB/NDS	wt	2/2	5
NTHL1	***16***	c.527T>C p.(Ile176Thr)	1/1	0/1	NP			3
	75	c.856G>A p.(Gly286Ser)			NP			3
	82	c.527T>C p.(Ile176Thr)			NP			3
POLD1	***116***	c.2007-5C>T	1/1	1/1	NP(+/-5)	wt		1
	118	c.520C>T p.(Arg174Trp)	2/3		NP			3
	152	c.2052G>C p.(Gln684His)			NP			3
	157	c.1465G>A p.(Val489Met)			NP			3
POLE	***16***	c.3857G>A p.(Arg1286His)	1/1	0/1	NP			3
	21	c.6716C>T p.(Ala2239Val)	1/2	1/1	NP			2
	147	c.198G>A p.(Met66Ile)			NP			3

[a]Affected:number of affected carrier members/total of affected members.[b]Healthy:number of healthy carrier members/total of healthy members. [c]Prediction: *In silico* predictions by programs Human Splicing Finder (HSF) and MaxEnt[1]. NP= not predicted alteration, +/-5= variants located between nucleotides +/-5 of the intron/exon boundaries, DB= donor broken prediction (HSF CV=-95.4% and MaxEnt CV=-411.44%); NDS=new donor site prediction (HSF CV=+1355.6% and MaxEnt CV=+144,49%); [d]G>T somatic changes= number of G>T detected changes / number of total somatic changes. [e]CLASS: variant classification: 1= benign; 2=likely benign; 3=uncertain significance; 4= likely pathogenic; 5= pathogenic.Reference sequences: *APC*: NM_000038.4, NP_000029.2; *AXIN2*: NM_004655.3, NP_004646; *MUTYH*: NM_001128425, NP_001121897; *NTHL1*: NM_002528, NP_002519; *POLD1*: NM_001256849, NP_001243778; *POLE*: NM_006231, NP_006222.

Supplementary table 3B. Reclassified variants following ACMG-SHERLOC criteria[2]**.**

RECLASSIFIED VARIANTS				
**HGVS designation**	**c.739C>G p.(Arg247Gly)**	**c.1510_1517delins p.Thr504Profs*68**	**c.2007-5C>T**	**c.6716C>T p.(Ala2239Val)**
**GENE**	*MUTYH*	*MUTYH*	*POLD1*	*POLE*
**ID**	35	89	116	21
**SHERLOC EVIDENCE TYPES**[a]				
**Population: Frequency and Homozygotes**	EV0135 (1P)	EV0135 (1P)	EV0161 (1B)	EV0161 (1B)
**Observation in Affecteds**	EV0154(1.5P)/EV0156(1P)	EV0154(1.5P)/EV0156(1P)		-
**Co-ocurrence in Affected**		-	-	-
**Observations in Unaffected**	-	-	EV0053 (2B)	EV0084 (2B)
**Variant Effect**		EV017<br>83 (2P)		
**Functional Experiments**	-	-	EV0036 (2.5B)	-
**Lab Assays**	-	-	-	-
**Computational &Predictive**[a]	EV0122 (0,5P)	EV0122 (0,5P)	-	EV0122 (0.5P)
**POINTS**	4P	5P	5.5B	3B/0.5P
**CLASS**[b]	**4**	**5**	**1**	**2**

[a]Sherloc evidence types: EV=evidence number; P=points of pathogenic evidence; B=points of benign evidence. [b]CLASS: variant classification: 1= benign; 2=likely benign; 3=uncertain significance; 4= likely pathogenic; 5= pathogenic. Pathogenic and benign evidences were scored separately and variants were classified into 5 groups according to total score: class 5=variants with at least 5 points of pathogenic evidences; class 4= variants with 4 to 5 points of pathogenic evidences; class 2= variants with 3 to 5 points of benign evidences; class 1= variants with at least 5 points of benign evidences; class 3= all remaining variants.

1. Desmet, F. O. *et al.*Human Splicing Finder: an online bioinformatics tool to predict splicing signals. *Nucleic Acids Res***37**, e67 (2009).
2. Nykamp, K. *et al.* Sherloc: a comprehensive refinement of the ACMG-AMP variant classification criteria. *Genet Med***19**, 1105–1117 (2017).

Supplementary figure 1. Allele-specific amplification for biallelic*MUTYH* mutations.

A

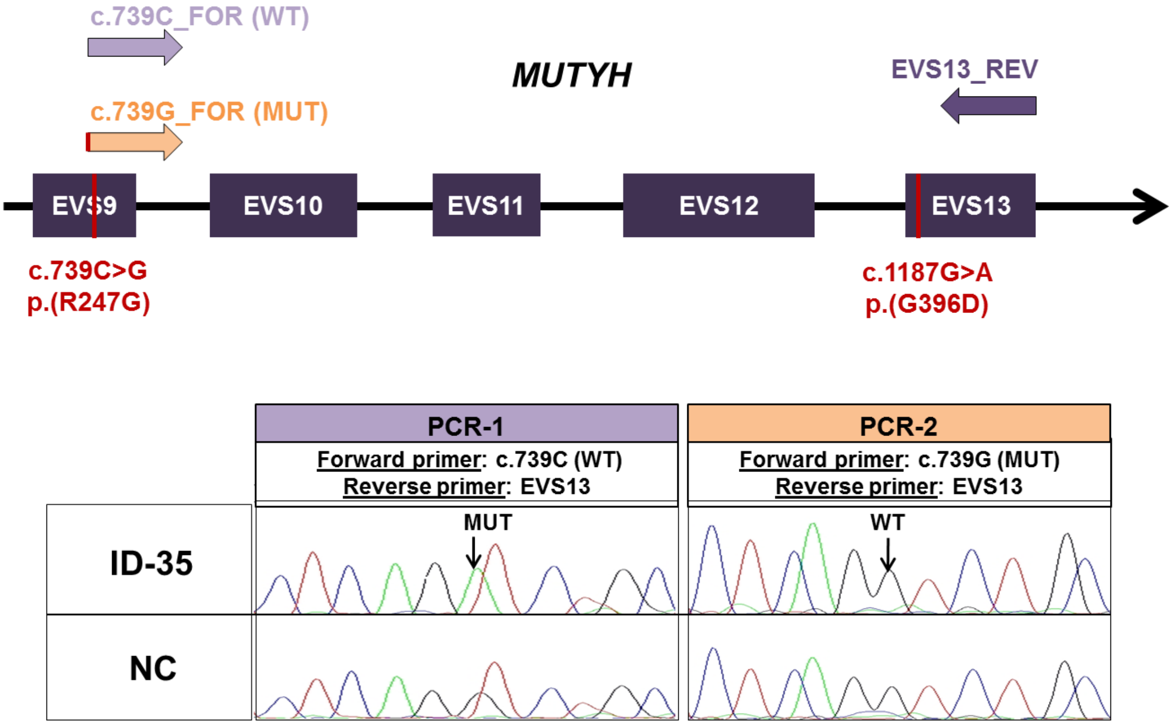

B

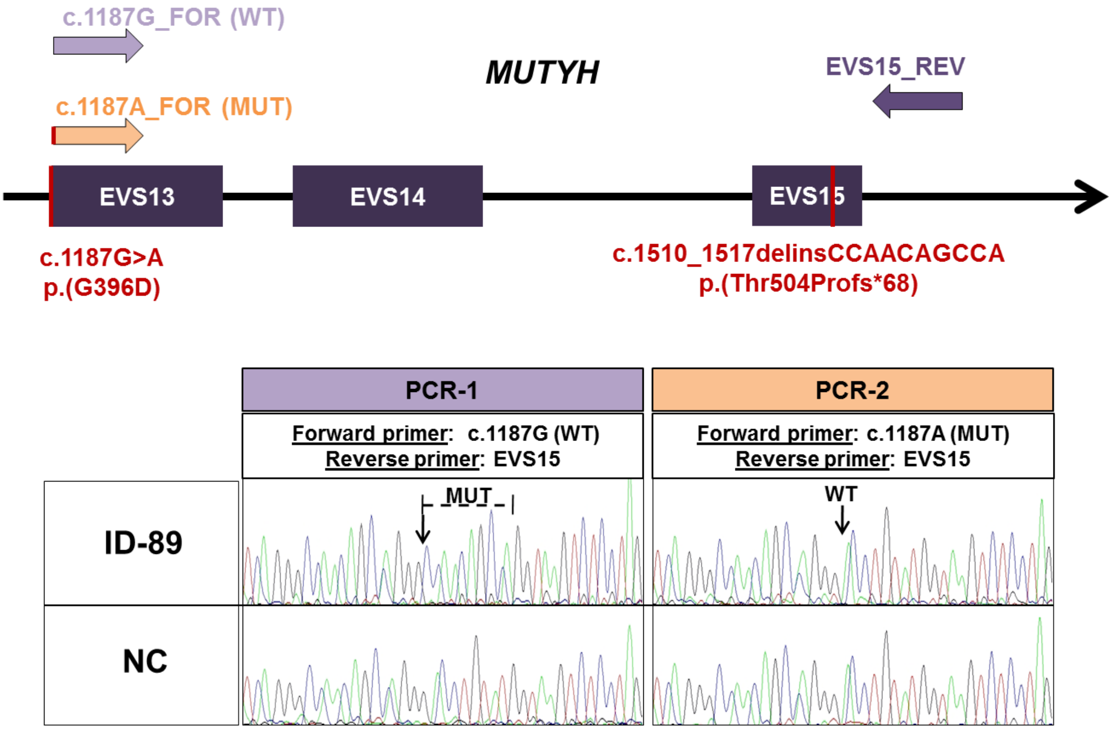

Two *MUTYH* non-recurrent mutations, c.739C>G and c.1510_1517delinsCCAACAGCCA, were found in co-occurrence with the recurrent mutation c.1187G>A. To check if concurrent mutations were located in TRANS, allele-specific amplifications and subsequent sequencing were performed in both cases. **A)** PCR-1: c.739C>G wild-type allele (C) was amplified with the specific forward primer c.739C_FOR and the reverse primer EVS13_REV which was located downstream the position of the second mutation c.1187G>A. After Sanger sequencing of the corresponding amplification product, c.1187G>A mutant allele (A) was detected. PCR-2: c.739C>Gmutant allele (G) was amplified with the specific forward primer c.739G_FOR and the reverse primer EVS13_REV. After Sanger sequencing of the corresponding amplification product, c.1187G>A wild-type allele (G) was detected. Therefore, c.739C>G and c.1187G>A are located in different alleles (TRANS). **B)** PCR-1: c.1187G>A wild-type allele (G) was amplified with the specific forward primer c.1187G_FOR and the reverse primer EVS15_REV which was located downstream the position of the second mutation c.1510_1517delinsCCAACAGCCA. After Sanger sequencing of the corresponding amplification product, c.1510_1517delinsCCAACAGCCAmutant allele was detected. PCR-2: c.1187G>Amutant allele (A) was amplified with the specific forward primer c.1187A_FOR and the reverse primer EVS15_REV. After Sanger sequencing of the corresponding amplification product, c.1510_1517delinsCCAACAGCCA wild-type allele was detected. Therefore, c.1510_1517delinsCCAACAGCCA and c.1187G>A are located in different alleles (TRANS). FOR= Forward, REV= Reverse, NC= Negative control, WT: wild type, MUT: mutant.

Supplementary figure 2. cDNA/gDNA amplification after DNAse treatment in *POLE/NTHL1* carrier.

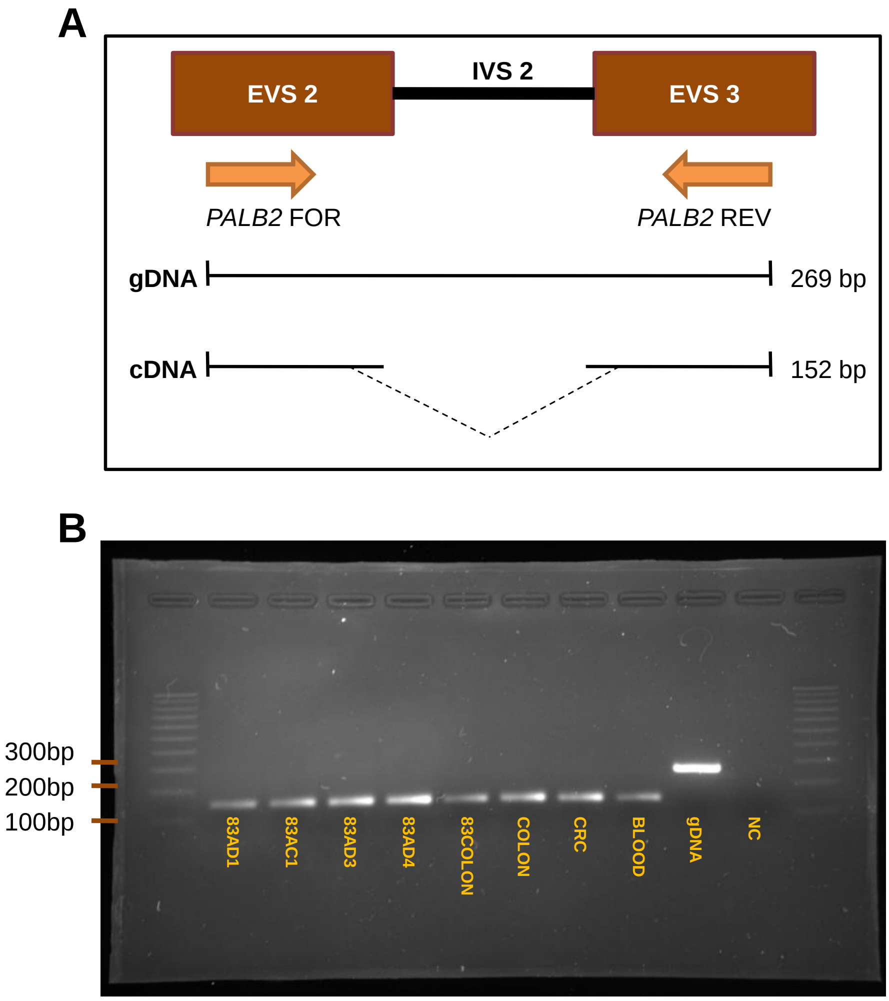

The lack of germline DNA traces was checked by differential germline and cDNA amplification. ***A.***Amplicon design between two different exons; genomic DNA amplification gives rise to a fragment of 269pb; cDNA amplification gives rise to a fragment of 152pb, which excludes the intron sequence. **B.** Agarose gel electrophoresis of DNAse-treated cDNA samples. 83AD1, AD3 and AD4= low dysplastic adenomas from carrier; AC1=adenocarcinoma from carrier; 83COLON= healthy colon tissue from carrier; COLON= healthy colon control pool; CRC= colorectal cancer control pool;gDNA = control genomic DNA; NC= negative control.No cDNA sample showed gDNA amplification.
